# Differential mast cell numbers and characteristics in human tuberculosis pulmonary lesions

**DOI:** 10.1038/s41598-021-89659-6

**Published:** 2021-05-21

**Authors:** Karen Magdalena Garcia-Rodriguez, Estela Isabel Bini, Armando Gamboa-Domínguez, Clara Inés Espitia-Pinzón, Sara Huerta-Yepez, Silvia Bulfone-Paus, Rogelio Hernández-Pando

**Affiliations:** 1 Lydia Becker Institute of Immunology and Inflammation, Faculty of Biology, Medicine and Health, University of Manchester, Manchester, UK; 2 Manchester Collaborative Centre for Inflammation Research, Faculty of Biology, Medicine and Health, University of Manchester , Manchester, UK; 3 Seccion de Patologia Experimental, Instituto Nacional de Ciencias Medicas y Nutricion “Salvador Zubiran”, Mexico City, Mexico; 4 Departamento de Inmunologia, Instituto de Investigaciones Biomedicas, Universidad Nacional Autonoma de Mexico, Mexico City, Mexico; 5 Division of Musculoskeletal and Dermatological Sciences, Faculty of Biology, Medicine and Health, University of Manchester, Manchester, UK; 6 Unidad de Investigacion en Enfermedades Oncologicas, Hospital Infantil de Mexico, Federico Gomez, Mexico City, Mexico

**Keywords:** Microbiology, Immunology, Infection, Infectious diseases, Inflammation

## Abstract

Tuberculosis (TB) is still a major worldwide health threat and primarily a lung disease. The innate immune response against *Mycobacterium tuberculosis* (*Mtb*) is orchestrated by dendritic cells, macrophages, neutrophils, natural killer cells and apparently mast cells (MCs). MCs are located at mucosal sites including the lungs and contribute in host-defence against pathogens, but little is known about their role during *Mtb* infection. This study investigates the location and characteristics of MCs in TB lesions to assess their contribution to TB pathology. To this purpose, number, location and phenotype of MCs was studied in 11 necropsies of pulmonary TB and 3 necropsies of non-TB infected lungs that were used as controls. MCs were localised at pneumonic areas, in the granuloma periphery and particularly abundant in fibrotic tissue. Furthermore, MCs displayed intracellular *Mtb* and IL-17A and TGF-β immunostaining. These findings were validated by analysing, post-mortem lung tissue microarrays from 44 individuals with pulmonary TB and 25 control subjects. In affected lungs, increased numbers of MCs expressing intracellularly both tryptase and chymase were found at fibrotic sites. Altogether, our data suggest that MCs are recruited at the inflammatory site and that actively produce immune mediators such as proteases and TGF-β that may be contributing to late fibrosis in TB lesions.

## Introduction

Tuberculosis (TB) caused by *Mycobacterium tuberculosis* (*Mtb*), remains one of the deadliest bacterial infections worldwide^1^. During infection, *Mtb* reaches the lungs where different innate immune cells reside including mast cells (MCs)^2–4^. *Mtb* is phagocytosed by macrophages leading to the release of diverse cytokines, including TNFα and IL-6 that drive the inflammatory process^5^. To control *Mtb* spread, innate and adaptive immune cells surround infected phagocytic cells promoting granuloma formation^5,6^. The cytokines IL-17A and TNF-α are known to contribute to the process^7^. When granuloma containment fails, different lung injuries including pneumonia, bronchitis, caseous necrosis and eventually fibrosis prevail^8,9^. The fibrotic process in human lungs has been associated with the presence of TGF-β, and the proteases, tryptase and chymase^10,11^. The final TB pathology phase culminates in irreversible lung tissue damage manifested by necrosis and fibrosis^8,9^. Although many cells are involved in this process, little is known about the contribution of MCs in this pathology.

MCs are distributed in lungs and mucosal tissues and contribute to host-defence against bacterial infections^12^. In humans, MCs are classified as tryptase + MCs (MC_T_), chymase + MCs (MC_C_) or both tryptase + and chymase + MCs (MC_TC_)^13^. Upon bacterial exposure, they release a wide variety of cytokines and chemokines, including IL-17, TNF-α, IL-8 and TGF-β either by degranulation or canonical secretory pathways^14^. Additionally, during lung infections MCs are altered in numbers, phenotype and localization^15,16^. For instance, MC numbers are decreased in lungs of *Streptococcus pneumoniae* infected patients^17^. Furthermore, MCs are capable to phagocytose bacteria and present antigens^18^. Although hypothetically MCs have an important role in TB^3^, little has been explored. For instance, it is unknown if MCs are localized at pulmonary tuberculous human lesions and what is the predominant MC phenotype. It is also unclear whether MC cytokines, e.g. IL-17A, and TGF-β contribute to the inflammatory process, and formation and maintenance of granulomas and fibrogenesis.

In this study we examined number, distribution and phenotype of MCs in human TB-infected lungs and MC cytokine expression at granulomas and fibrotic sites in TB-infected lung tissue. Our descriptive findings demonstrated that MCs are likely to participate to the early inflammatory phase, as well as to the late fibrosis formation during TB pathology.

## Materials and methods

### Ethical statement and tissue procurement

All methods were performed in accordance to relevant guidelines and regulations. Lung tissue sections from 11 necropsies of deceased TB patients and 3 controls from non-TB infected necropsies were obtained from the Pathology Department files at the National Institute of Medical Sciences and Nutrition “Salvador Zubiran” in Mexico City. A specific informed consent was not required for this study, but every medical autopsy was performed with a signed authorization of the representative legal family member and tissue samples were obtained during legally authorized autopsies with signed permission by a relative who agreed tissue sample donation for the present and previous studies^19^. The microarray tissue study from 44 individuals with pulmonary TB and 25 control subjects were taken from the Pathology Department of the General Hospital of Mexico ‘Eduardo Liceaga’ at Mexico City, and ethical statement was approved by the local ethic committee of the Hospital Infantil de Mexico, Federico Gomez (No. HIM/2008/015). This study including all experimental protocols were approved by the in-house ethical committee at the National Institute of Medical Sciences and Nutrition “Salvador Zubiran”.

### TB tissue processing

Macroscopically, lung tissue from 11 necropsies of deceased TB patients showed extensive cavitary bilateral lung disease, surrounded by numerous white nodules with irregular shapes and size that alternated with pneumonia patches. Extensive sampling was performed, obtaining several tissue fragments from different lesions and were embedded in paraffin blocks sectioned at 3 µm and mounted in glass slides. Additionally, we used post-mortem lung tissue, essentially granulomas, from 44 individuals with pulmonary TB and 25 control subjects (subjects who died as a result of any other cause without significant pulmonary disease). The lung tissues (TB and control tissue samples) were organized in a tissue microarray (MTA) as previously described^20^. One sample of each section (lung sections and MTA) were stained with haematoxylin and eosin (HE) to select lung lesions for study. Spare sections were left at room temperature before being used for immunoperoxidase and immunofluorescence staining. The lung tissues in this study correspond to active TB cases, not latent TB cases were included.

### Immunoperoxidase staining

Lung sections were deparaffinized and treated with antigen retriever (1X; Bio SB, Santa Barbara, California) for 5 min under microwave heating. Endogenous peroxidase was blocked incubating tissue with methanol-H2O2 (9:1) for 10 min. After three washes, unspecific sites were blocked using a background sniper (BIOCARE MEDICAL; Pacheco, California) for 30 min. Slides were washed and incubated with either a rabbit anti-human chymase antibody (Ab186417, Abcam; Cambridge, United Kingdom) or a mouse anti-tryptase antibody (Ab2378, Abcam; Cambridge, United Kingdom) for 2 h. After three washes, tissue was processed using a mouse/rabbit PolyDetector DAB (3–3′-diaminobenzidine)/HRP (horseradish peroxidase) brown detection system (BSB0219, Bio SB; Santa Barbara, California) following manufacturer´s instructions. Micrographs were acquired using a LEICA DMLS microscope with a 2.5X and 40X dry objectives equipped with a LEICA DFC295 camera and analysed using an automated image analyser (QWin Leica; Wetzlar, Germany).

### Immunofluorescence staining

To visualize MC phenotypes, lung tissue sections were deparaffinized, treated with DNA retriever (1X; Bio SB; Santa Barbara, California) for 5 min under heating and incubated with blocking buffer (goat serum 1:10 in PBS + tween 0.1%) for 30 min. After three washes, tissue were incubated with a rabbit anti-human chymase antibody (Ab186417, Abcam; Cambridge, United Kingdom), and a mouse anti-tryptase antibody (Ab2378, Abcam; Cambridge, United Kingdom) for 1 h followed by 1 h incubation with a goat anti-mouse antibody conjugated to Alexa 488 fluorophore (Ab150117, Abcam; Cambridge, United Kingdom) and a goat anti-rabbit antibody conjugated to Alexa 647 fluorophore (Ab150083, Abcam; Cambridge, United Kingdom). After three washes, tissue was mounted using a fluoroshield mounting media containing 4′,6-diamidino-2-phenylindole (DAPI, Abcam; Cambridge, United Kingdom). Slides were analysed using a fluorescent microscope OlympusBX41 with either 40 × and 10 × dry objectives. Images were acquired using a Zen 2.6 blue and analysed using Fiji.

To analyse cytokine expression and *Mtb* internalization, lung sections were deparaffinized, treated with DNA retriever (1X; Bio SB; Santa Barbara, California) for 5 min under heating and incubated with blocking buffer (goat serum 1:10 in PBS + tween 0.1%) for 30 min. After three washes, tissues were incubated with a mouse anti-tryptase antibody (Ab2378, Abcam, Cambridge, United Kingdom) and either a rabbit polyclonal anti-*Mtb* (CP140C, BioCare Medical), capable to recognize diverse *Mtb*-cell wall and secreted antigens, rabbit anti-TGF-β (Jackson Immunoresearch, Cambridge, United Kingdom) or rabbit anti-IL-17 (SC7927 Santa Cruz Lab, USA) antibodies for 1 h followed by 1 h incubation with a goat anti-mouse antibody conjugated to either Alexa 488 (Ab150117, Abcam; Cambridge, United Kingdom) or Alexa 647 fluorophores (Ab150115, Abcam; Cambridge, United Kingdom) and a goat anti-rabbit antibody conjugated to either Alexa 647 (Ab150083, Abcam; Cambridge, United Kingdom) or Alexa 488 fluorophores (Ab150081, Abcam; Cambridge, United Kingdom). After three washes, tissue was mounted using a fluoroshield mounting media containing 4′,6-diamidino-2-phenylindole (DAPI, Abcam; Cambridge, United Kingdom). Slides were analysed using a confocal microscope LSM 710 DUO, Carl Zeiss.

### MC quantification

Forty-four MTAs from TB-infected lung sections from autopsy cases and 22 non-TB infected controls contained in 4 different slides were stained with HE and analysed. Five MTAs were selected as control lung tissue and 10 MTAs presenting fibrosis were selected as representative fibrotic tissue. Selected MTAs were immunostained with tryptase and chymase (as described above) and studied at 10 × magnification using a fluorescent microscope OlympusBX41 and acquired using Zen 2.6 blue software system. One high power field was taken for each MTA and all single positive (MC_C_ or MC_T_) and double positive (MC_TC_) cells were counted per field using Fiji. MC numbers were graphed using GraphPrism.

### Statistical analysis

A Shapiro–Wilk tests was performed to determine normality during phenotype quantification. Statistical analysis was achieved using Kruskal–Wallis test and a Dunn’s multiple comparison post-test (adjusted *p* ≤ 0.01) using GraphPad Prism 8th edition.

### Compliance with Ethical standards

Authors declare that no conflict of interest happened during the present study. The present research involved the use of lung sections obtained from deceased individuals. Autopsies were selected from the Pathology Department files at the National Institute of Medical Sciences and Nutrition “Salvador Zubiran” in Mexico City. A specific informed consent was not required for this study, but every medical autopsy was performed with a signed authorization of the representative legal family member and tissue samples were obtained during legally authorized autopsies with signed permission by a relative who agreed tissue sample donation for this and previous studies^19^. Additional tissue was obtained from the Pathology Department of the General Hospital of Mexico ‘Eduardo Liceaga’ at Mexico City, and ethical statement was approved by the local ethic committee of the Hospital Infantil de Mexico, Federico Gomez (No. HIM/2008/015).

### Experimental procedures

All methods were performed in accordance to relevant guidelines and regulations. This study including all experimental protocols were approved by the in-house ethical committee at the National Institute of Medical Sciences and Nutrition “Salvador Zubiran”.

## Results

### Tryptase positive mast cells are the most abundant phenotype in non-TB infected human lungs

**Figure 1:**
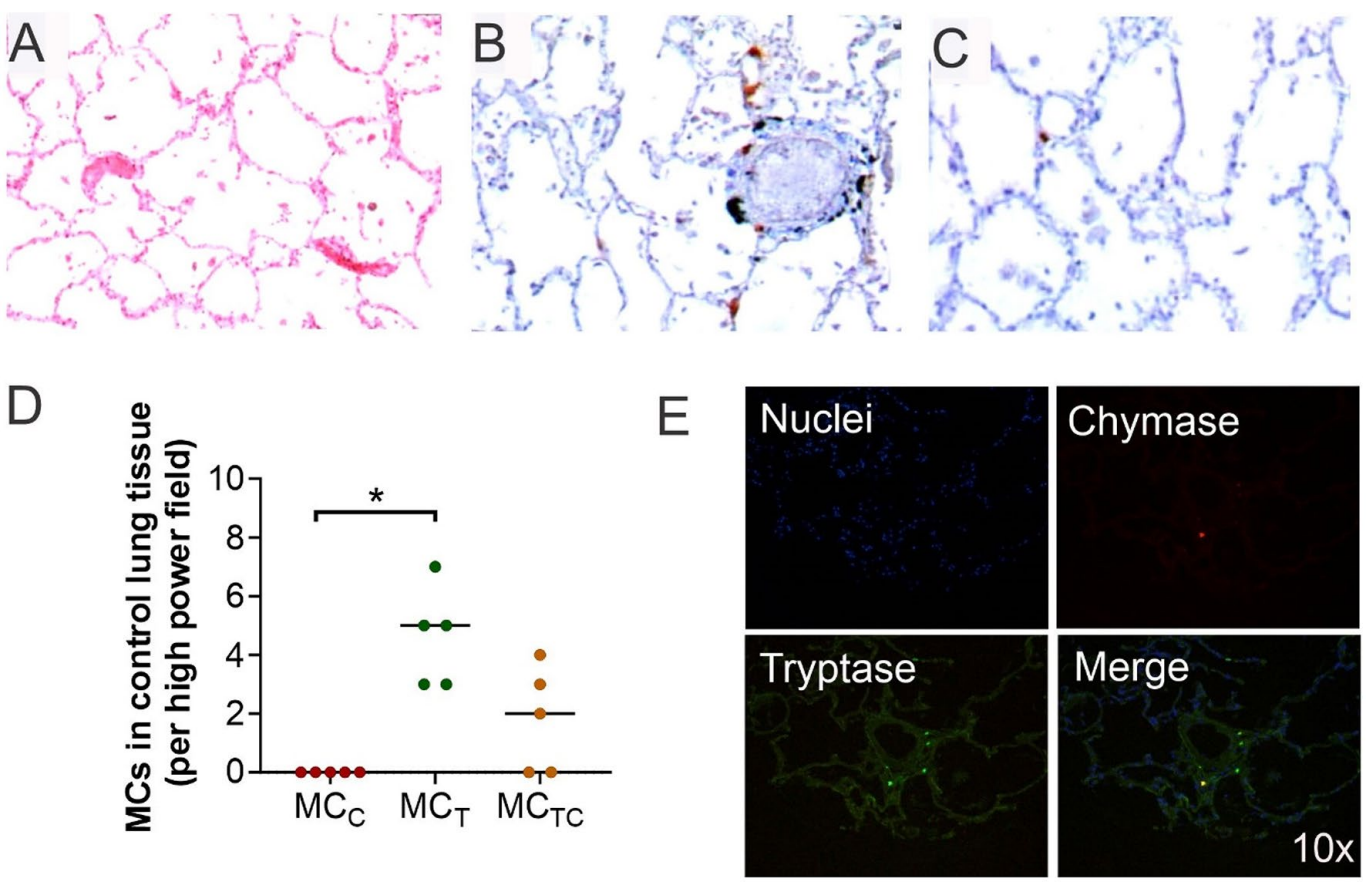
Tryptase positive mast cells are predominant in non-tuberculosis infected human lung tissue. (**A**) Representative micrograph HE staining of lung section from non-TB infected lung autopsy (cause of death heart attack) showing preserved alveolar structure. (**B**) Tryptase Immunostaining shows several positive MCs in the alveolar-capillary interstitium and in the adventitia layer of blood vessels. (**C**) Chymase immunostaining shows occasional positive cells. (**D**) Predominance of tryptase + MCs in non-tuberculous lung tissue was analysed by morphometry in five microarrays. (**E**) Representative immunofluorescence microarray studied with fluorescent microscopy shows MC_T_ (green), MC_C_ (red) and MC_TC_ (merge) surrounding a blood vessel. Shapiro–Wilk test was done to determine normality. Statistical comparison was performed using Kruskal–Wallis test and Dunn´s multiple comparison post-test (adjusted p ≤ 0.01).

In physiological conditions MCs expressing either tryptase or chymase or both proteases reside in alveolar parenchyma. However, in pulmonary infections MC numbers and phenotype are altered^21^. To investigate lung MC distribution and their characteristics we studied their number, location and phenotype in autopsies from control lungs (non-TB infected). Control cases had heart attacks as death cause with lungs showing overall a normal structure with some focal patches of centrilobular emphysema (Fig. 1A). These tissues showed MC_T_ (Fig. 1B) and lesser MC_C_ (Fig. 1C) at alveolar walls. Both MC_T_ and MC_C_ were preferentially located in blood vessels adventitia. Moreover, MC_T_ were the most abundant phenotype (median = 5 cells per field) (Fig. 1D) followed by MC_TC_ (median = 2 cells per field), whereas MC_C_ were not detected. In fact, all MC_C_ observed were also tryptase + therefore MC_TC_ (Fig. 1E). Thus, MCs expressing only chymase were rare whereas tryptase positive MCs were predominant in human lung parenchyma.

### Mast cells are located at active inflammatory sites of TB-infected lungs and show intracellular mycobacterial antigens

**Figure 2:**
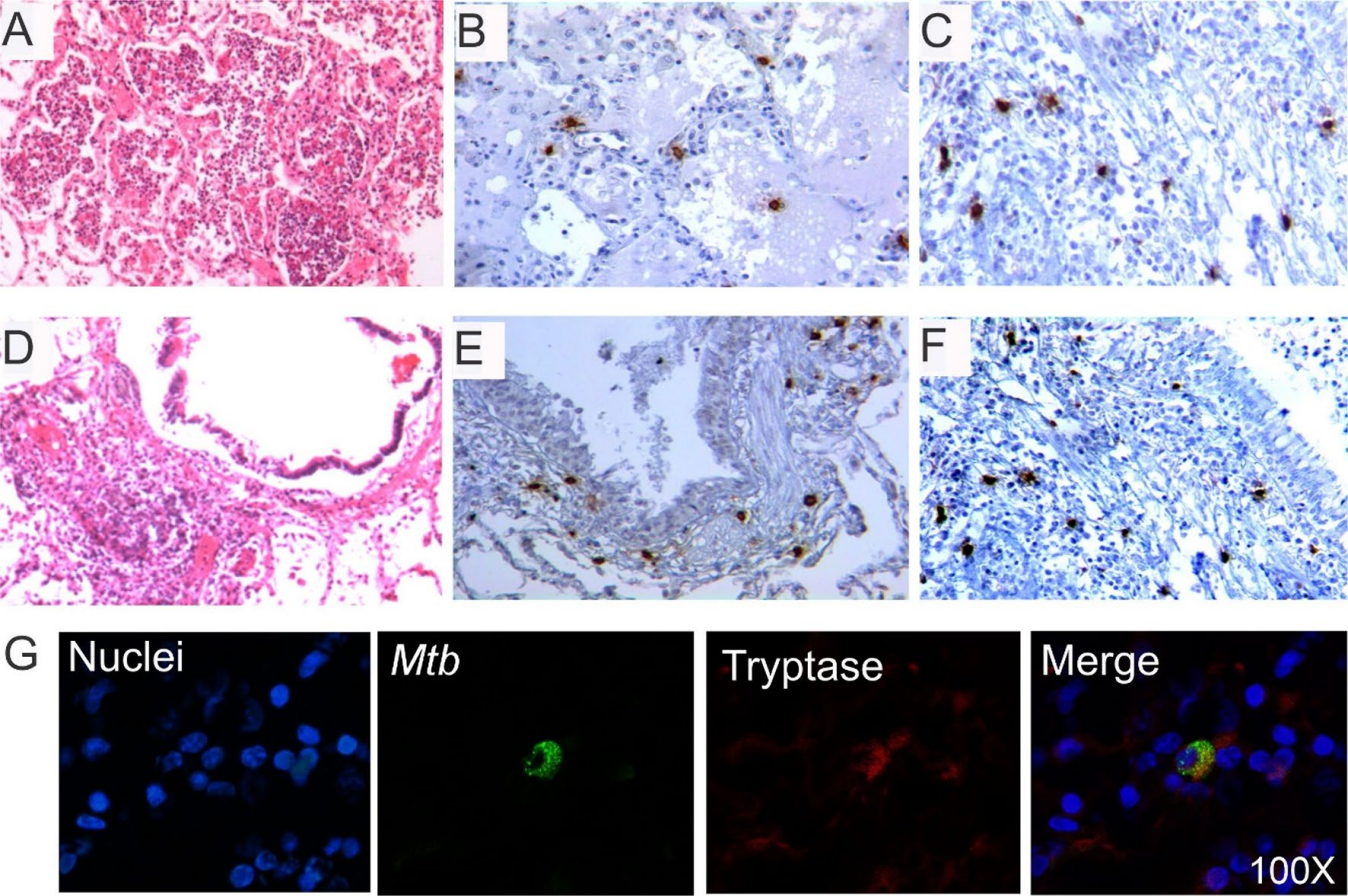
Tryptase and chymase positive mast cells are located at active inflammatory lesions and show intracellular mycobacterial antigens. (**A**) Representative HE micrograph showing areas of tuberculous pneumonia. (**B**) Tryptase + MCs are present in alveolar walls and lumen. (**C**) Chymase + MCs show similar numbers and distribution as tryptase + MCs in the pneumonic areas. (**D**) Extensive inflammatory infiltrate is detected in bronchial walls. (**E**) Numerous tryptase + MCs are located in the bronchial submucosa. (**F**) Numerous chymase + MCs are in bronchial walls and neighbour alveoli. Micrographs are representative of 11 necropsies of TB patients. (**G**) A representative section with extensive inflammation in pneumonic areas was incubated with polyclonal anti-*Mtb* (Alexa 488 label) and anti-tryptase antibodies (Alexa 647 label). High power micrograph shows a MC expressing tryptase that colocalize with *Mtb* antigens.

MC numbers, location and phenotype found in non-infected lungs were used as controls to investigate MC characteristics in TB lung lesions. A table summarizing clinical data from the studied TB active necropsies is shown in Supplemental Table 1. Both MC phenotypes (MC_T_ and MC_C_) were abundant at inflammatory (granulomas, pneumonia, vascular and airways walls) and fibrotic areas (Fig. 2), but absent in the proximity of necrotic sites (Supplemental Fig. 1). At pneumonic areas (Fig. 2A), numerous MCs were seen in alveolar walls and alveolar lumen (Fig. 2B,C), while in blood vessels (Fig. 2D) MCs were found in the adventitia and in bronchial airways (Fig. 2E,F). Both phenotypes were positioned below the epithelium, in the submucosa and in the muscular wall between smooth muscle cells. Furthermore, as shown in Fig. 2G, MC_T_ stationed in inflammatory regions of TB-infected lung sections showed vacuoles containing *Mtb* antigens. Thus, both MC_T_ and MC_C_ reside in TB lung lesions and store *Mtb*.

### Mast cells are located at the periphery of granulomas and express IL-17

**Figure 3:**
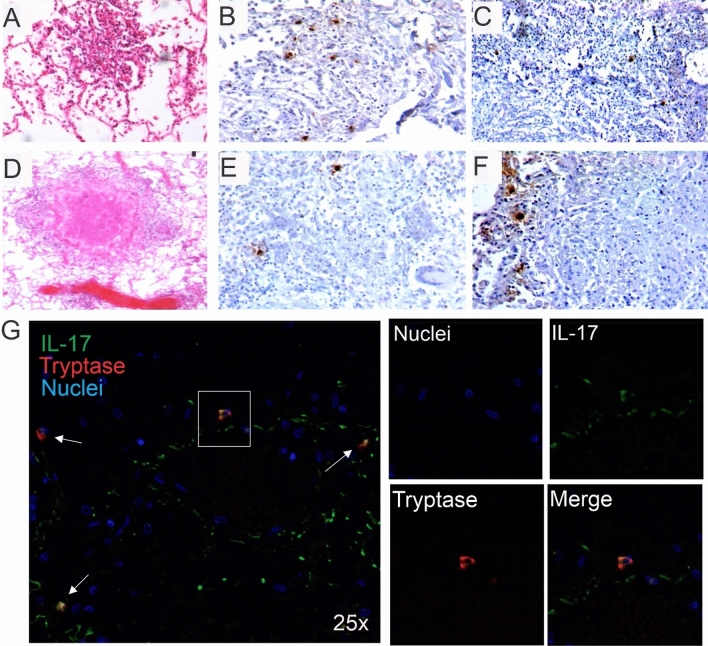
Mast cells are observed in granulomas and express IL-17A. Representative micrographs of different types of granulomatous lesions (incipient, mature or necrotic) were stained with HE or with anti-human anti-tryptase or anti-chymase immunoperoxidase antibodies to identify MC_T_ or MC_C_. (**A**) Small incipient granuloma. Both MC phenotypes (**B**) MC_T_ and (**C**) MC_C_ are detected within the inflammatory area of incipient granulomas. **(D)** Mature granuloma with central necrosis. (**E**) MC_T_ and MC_C_ (**F**) are not infiltrating mature granulomas but both subtypes are observed in the periphery. Micrographs are representative of 11 TB necropsies. Representative section of incipient granuloma was incubated with anti-IL-17A (Alexa 488 label) and anti-tryptase antibodies (Alexa 647 label). (**G**) Low power micrograph shows numerous IL-17 + cells, and MC_T_. High power micrograph of the inset shows MC_T_ staining positive for IL-17A.

Granulomas are characterized by a necrotic core containing *Mtb* surrounded by macrophages and lymphocytes and a fibrotic external layer^20^. As shown in Fig. 3, granulomas at different stages were analysed. In early or incipient granulomas (Fig. 3A), characterized by small nodular congregates of inflammatory cells, occasional MCs were seen intermixed with lymphocytes and macrophages (Fig. 3B,C). However, MCs were not observed to infiltrate typical or mature well-organized granulomas (Fig. 3D) but were located at their periphery (Fig. 3E,F). Indeed, MCs were abundant at the fibrotic outer layer of necrotic granulomas (Fig. 3F). Representative TB-infected tissue containing typical granulomas or incipient granulomas was incubated with anti-IL-17A and anti-tryptase antibodies followed by a fluorescent staining. As shown in Fig. 3G, IL-17 positive MCs were observed at neighbour inflammatory tissue near to typical granulomas or mixed with inflammatory cells in incipient granulomas. Thus, MCs are virtually absent inside mature granulomas but located at their periphery and expressing IL-17.

### Mast cells are in high numbers in fibrotic tissue that surrounds granulomas and cavitary lesions

**Figure 4:**
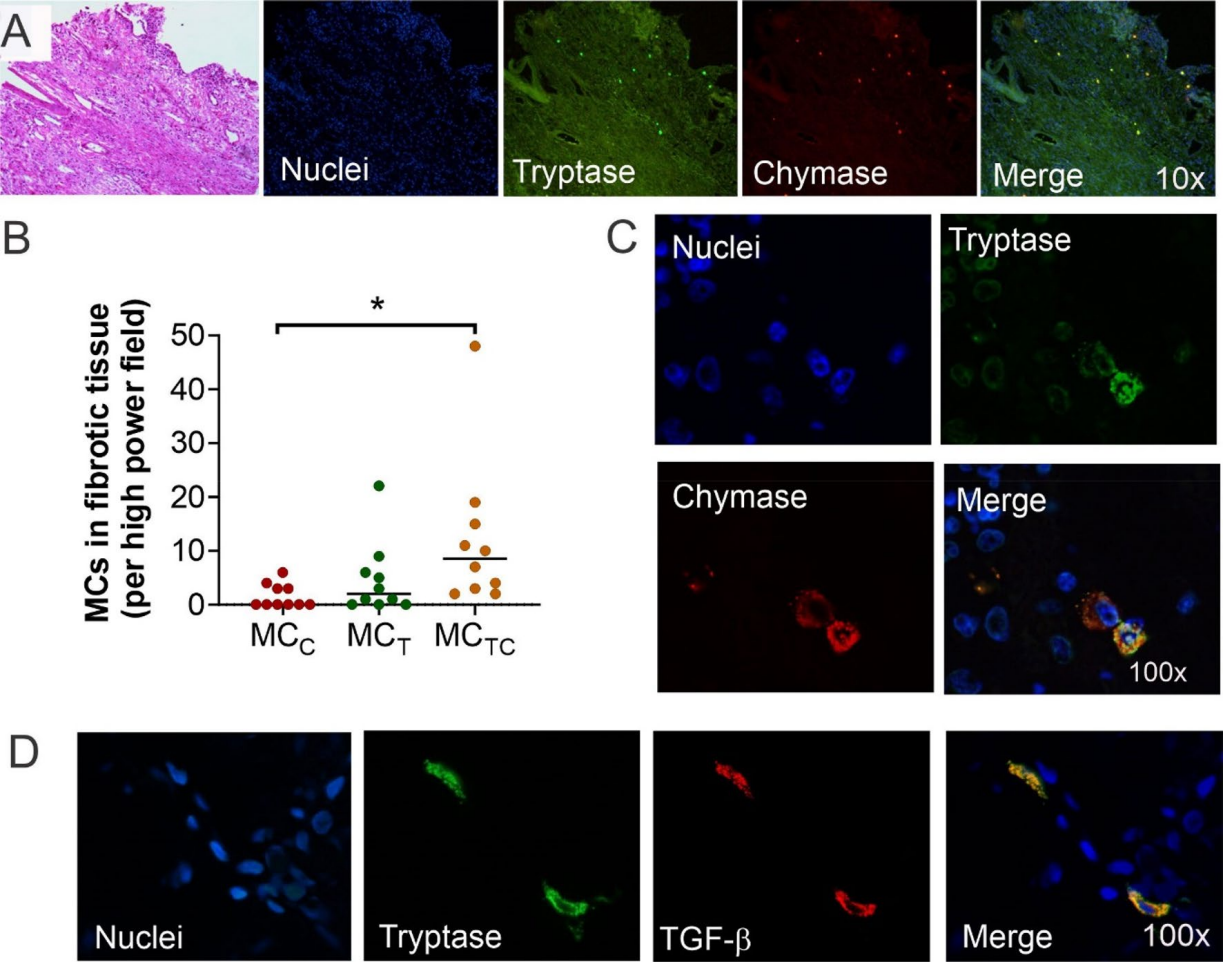
Tryptase and chymase positive mast cells are numerous in fibrotic lesions of tuberculous lungs. (**A**) Representative low power micrograph of fibrotic tuberculous nodule stained with HE and immunofluorescence micrographs from the same lesion shows MC_T_ (green), MC_C_ (red) and MC_TC_ (merge). (**B**) MCs subtypes counted in ten different high-power fields (microarrays) (40 ×) from 44 autopsy cases confirm that MC_TC_ were the most abundant phenotype at fibrotic areas. (**C**) High power micrograph at fibrotic areas shows MCs that express tryptase and chymase cytoplasmic granules which are partially degranulated. Shapiro–Wilk test was done to determine normality. Representative section of a fibrotic area was incubated with anti-TGF-β (Alexa 647 label) and anti-tryptase antibodies (Alexa 488 label). (**D**) High power micrograph shows TGF-β colocalization with MC_T_. Statistical comparison was performed using Kruskal–Wallis test and Dunn´s multiple comparison post-test (adjusted *p ≤ 0.01).

MCs were constantly detected at fibrotic areas around granulomas or wall cavities supporting a number of studies that have shown MCs participate in the fibrotic process^22,23^. To characterize the nature of the MCs residing at fibrotic areas of TB infected tissues we determined their proteases expression. Although MC_T_, MC_C_ and MC_TC_ phenotypes were all seen in fibrotic areas (Fig. 4A), MC_TC_ were the most abundant (median = 8.5 cells per field), followed by MC_T_ (median = 2 cells per field) (Fig. 4B). Since non-TB infected lung controls are colonised by MC_T_ (Fig. 1D), our data suggests a switch in proteases expression with an increase of chymase at TB-induced healing and fibrotic sites. Furthermore, by a double immunofluorescence labelling for TGF-β and tryptase, we could demonstrate that some MCs in fibrotic tissue not only express TGF-β (Fig. 4D) but exhibit a conserved but disorganized granular content suggesting their activation or partial degranulation (Fig. 4C). Thus, MCs at TB-induced lung lesions show an increased expression of chymase and pro-fibrogenic TGF-β and both may contribute to the fibrotic process.

## Discussion

Although MCs may play a role in TB infection^3^, little has been explored and the available studies were performed in rodent models. Furthermore, considering the differences in MC numbers and phenotypes between mouse and human lungs it is unclear whether the described findings are relevant for human pathology^21^. Our study demonstrates that in TB-infected human lungs MCs are located at pneumonic areas, in proximity to granulomas and particularly abundant at fibrotic sites. Besides, we observed a MC colocalization with *Mtb* antigens indicating an intracellular localisation of the pathogen. In addition, MCs located in proximity to granulomatous lesions were found to express IL-17, while TGF-β positive MCs were found embedded in the fibrotic tissue.

Our observations in lung controls showing MCs surrounding blood vessels and throughout the alveolar-capillary interstitial tissue with MC_T_ as the most abundant population are in line with current evidence that shows MC_T_ as predominant phenotype in large airways including bronchial and alveolar, followed by MC_TC_ which are more common at blood vessels, whereas MC_C_ are rare or sporadically observed in human lungs^24,25^. Although it is not clearly understood yet, this suggests that MC_T_ reside in the alveolar parenchyma prone to activation or differentiation upon environmental stimulus^16^.

Since MCs release a wide variety of cytokines, chemokines and antimicrobial molecules, they are likely to be involved in TB pathogenesis at different stages^3^. For instance, since our findings show that MCs locate at inflammatory areas of TB-infected human lungs, here MCs may contribute to the initial TB inflammatory stage by releasing TNF-α, IL-6, MCP-1, IL-1β, GM-CSF and IL-8. Muñoz et al. support this concept by showing the release of MC-dependent TNF and IL-6 upon *Mtb* infection^26,27^. Furthermore, the ability of MCs to contribute to immune cell recruitment was observed in *Chlamydia pneumoniae* lung infection characterised by a MC-dependent cellular infiltration in the lung airways^12,28–30^. Instead, the appearance of MCs at incipient granulomas and surrounding mature granulomas suggests their contribution in orchestrating granuloma formation and maintenance potentially by the secretion of IL-17, IL-6, IL-8, MCP-1, IL-10, IFN-γ and IL-1β^31,32^. However, MC mediators including TGF-β, IL-33, chymase and IL-1β are known to induce excessive inflammation and fibrosis and have detrimental effects^31^. Thus, suppressing MC-mediators release, e.g.IL-1β and TGF-β may diminish MC activation^33^ as proposed in SARS-COV2 infection^34,35^ and serve to prevent TB fibrosis.

In our study, we observe that human-lung resident MCs display intracellular *Mtb* fragments. This is in line with reported data^26,27^ showing MCs able to uptake *Mtb* via membrane binding through lipid rafts and CD48 receptor engagement^27^. Although MCs are not professional phagocytic cells, they are known to phagocytose^36^ and kill bacteria via acidified vacuoles^37^. These findings would support the role of MCs in uptaking and eliminating *Mtb* through phagocytosis or serve as a reservoir of intracellular *Mtb* during the active phase of TB infection. Although is not within the scope of this study, further research is needed to investigate whether MCs contribute to T cell activation as described in other conditions^36,38,39^ via *Mtb*-antigen presentation.

*Mtb* persistence promotes an ongoing cellular recruitment^40^ that results in the formation of early granulomas^6,40^. The association between MCs and TB-induced granulomas is still controversial. A positive correlation between MCs and granuloma formation was observed in tuberculous lymphadenitis tissue^41^ but not in tuberculous liver tissue^42^. Our data demonstrate that MCs infiltrate incipient granulomas and locate at the periphery or close proximity in mature or necrotic granulomas. Since different immune cells including T lymphocytes are part of the granuloma structure, a direct interaction between MCs and T cells is likely to occur at this site. MC cytokines including IL-17, and TNF-α are necessary for granuloma maturation and maintenance in mouse TB infection^43^. Although MC-TNF-α association was not observed (data not shown), we showed IL-17A positive MCs in the periphery of granulomas. Using an IL-17A gene-knockout mouse model, Okamoto-Yoshida et al*.* reported that IL-17A is necessary for granuloma maturation with γδ T cells as the major IL-17 source^44^. Also, granuloma formation was impaired in an IL-17A-deficient mouse model of sarcoidosis^45^. Therefore, we would like to propose the concept that MCs contribute to the initial step of cellular recruitment at the infection site, remain outside the inflammatory core during the adaptive immune stage, and orchestrate granuloma maturation via IL-17 expression.

In severe and chronic TB infection, fibrosis is the result of excessive inflammation^11^. MCs are abundant in fibrotic sites in non-infectious lung diseases^46^, including idiopathic pulmonary fibrosis^47^ and cystic fibrosis^48^. In addition, MCs products such as the fibroblast growth factor 2 (FGF-2)^49^, prostaglandin E2 (PGE2)^50^, IL-1β^51^, TGF-β^10,52,53^, tryptase^22^^23,54,55^ and chymase^47,56,57^ are known to contribute to fibrogenesis^50,53,58^. Our study reproduces findings described in idiopathic pulmonary fibrosis where MCs are increased in numbers and are partially degranulated at fibrotic sites^46,59^. Furthermore, we found a switch in MC phenotype from MC_T_ to MC_TC_ in fibrotic areas. In line with this, Andersson et al., correlated high MC_TC_ numbers with lung function, tissue remodelling and TGF-β1 expression^48^, suggesting MC_TC_ as important fibrosis mediators. In addition, mucosal MCs (MC_T_) in coculture with fibroblast lead to differentiation of MC_T_ into connective tissue MCs (MC_TC_). This process is coupled with an increase in fibroblast proliferation and an enhanced collagen synthesis necessary for fibrosis generation^60^. Moreover, some antimicrobial drugs are known to activate MCs^61^ or induce differentiation^62^ however, none of the treatments used for the patients from the reported autopsies in this study seems to have a direct effect on MC activation or differentiation. Thus, phenotypic change from MC_T_ to MC_TC_ with an increase in chymase expression induced by *Mtb* active infection would initiate or promote fibrosis together with the release of additional fibrogenesis-specific molecules.

## Conclusions

In conclusion, our results demonstrate that MCs expressing IL-17 are localizing in TB-induced human lung injuries at inflammatory sites while TGF-β positive and chymase rich MCs are stationed in the proximity of mature granulomas and embedded in fibrotic tissue. Although this is a descriptive study, our data suggest that MCs probably contribute to both, early immune cellular recruitment via IL-17A release and late fibrosis formation with differentiated MC_T_ into MT_TC_.

## Supplementary Information

Supplementary Information.

## Data Availability

The datasets generated during and/or analysed during the current study are available from the corresponding author on reasonable request.

## Abbreviations

MCs: Mast cells
TB: Tuberculosis
MC_T_: Mast cells expressing tryptase
MC_C_: Mast cells expressing chymase
MC_TC_: Mast cells expressing both tryptase and chymase
Mtb: *Mycobacterium tuberculosis*
DC: Dendritic cells
NK cells: Natural killer cells
