# Supplementary material for: Differential mast cell numbers and characteristics in human tuberculosis pulmonary lesions
Source: Sci Rep. 2021 May 21;11:10687. doi: 10.1038/s41598-021-89659-6 (PMC8140073; doi:10.1038/s41598-021-89659-6)
Supplement: Supplementary file 1 — Supplementary Information. [file 41598_2021_89659_MOESM1_ESM.docx]

**Differential mast cell numbers and characteristics in human tuberculosis pulmonary lesions**

Karen Magdalena Garcia-Rodriguez^1^, Estela Isabel Bini^2^, Armando Gamboa-Domínguez^2^, Clara Inés Espitia-Pinzón^3^, Sara Huerta-Yepez^5^, Silvia Bulfone-Paus^1,4^, Rogelio Hernández-Pando^2^*

*^1^Lydia Becker Institute of Immunolog and Inflammation, Manchester Collaborative Centre for Inflammation Research, Faculty of Biology, Medicine and Health, Manchester, United Kingdom, ^2^Instituto Nacional de Ciencias Medicas y Nutricion “Salvador Zubiran”, Seccion de Patologia Experimental, Mexico City, Mexico ^3^Departamento de Inmunologia, Instituto de Investigaciones Biomedicas, Universidad Nacional Autonoma de Mexico, Mexico City Mexico**, , ^4^Division of Musculoskeletal and Dermatological Sciences, Faculty of Biology, Medicine and Health, University of Manchester, Manchester, United Kingdom. ^5^Unidad de Investigacion en Enfermedades Oncologicas, Hospital Infantil de Mexico, Federico Gomez, Mexico City, Mexico.*

***Correspondence autor:**Rogelio Hernández-Pando
Instituto Nacional de Ciencias Médicas y Nutrición “Salvador Zubirán”,
Sección de Patología Experimental,
Mexico City, Mexico
[rhdezpando@hotmail.com](mailto:rhdezpando@hotmail.com)

**SUPPLEMENTARY MATERIAL**

**Supplemental Material**

**Supplemental Figure**


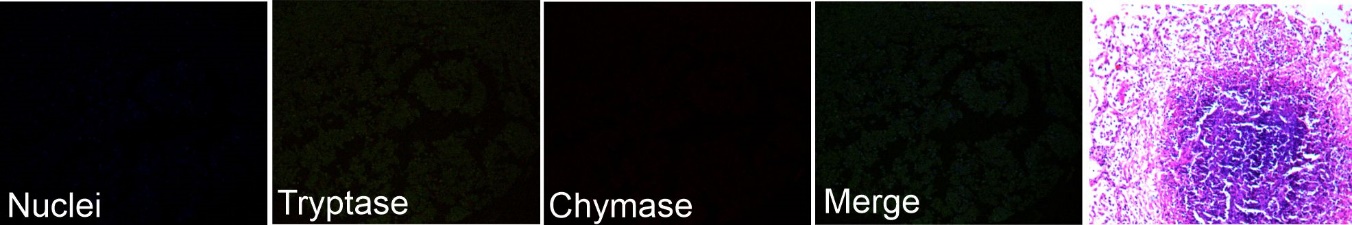


**Supplemental Figure 1**. **Mast cells are not present at necrotic sites of TB infected lungs.** 44 microarray sections from TB-infected patients and 22 from non-TB infected controls were stained with HE to visualize lung morphology to select necrotic tissue. Microarray sections with necrosis were incubated with anti-tryptase and anti-chymase antibodies followed by their fluorescent staining. Figure shows 2 representative micrographs of necrotic tissue in the absence of MC_T_ (green), MC_C_ (red) and MC_TC_ (merge) at necrotic sites of TB-infected lungs.

**Supplemental Table**

| **Gender** | **Age (years)** | **Cause of death** | **Treatment** |
| --- | --- | --- | --- |
| Male | 20 | Disseminated TB | Clarithromycin, Ampicillin, isoniazid, rifampicin, ethambutol. |
| Male | 56 | Cavitated pulmonary TB | Isoniazid, rifampicin, ethambutol, pyrazinamide, Imodium |
| Male | 61 | Disseminated TB | isoniazid, rifampicin, ethambutol, pyrazinamide, methyl-dopa propranolol |
| Male | 70 | Pulmonary, ganglionar TB | gentamicin, tetracyclines, ampicillin, amikacin, clindamycin |
| Male | 44 | Disseminated TB | Ampicillin, neomycin |
| Female | 63 | Disseminated TB | Methyldopa, furosemide |
| Female | 75 | Cavitated pulmonary TB | Ampicillin, gentamicin |
| Male | 38 | Disseminated TB, hepatitis induced by isoniazid | Isoniazid, rifampicin, ethambutol, pyrazinamide. |
| Male | 34 | Disseminated and cavitated TB | Isoniazid, rifampicin, ethambutol, pyrazinamide |
| Male | 25 | Extensive cavitated TB | Spironolactone, neomycin |
| Female | 86 | Cavitated, nodular TB | Methyldopa, spironolactone |

**Supplemental Table 1**. **Clinical data from necropsies of deceased TB patients.** For this study, lung tissue sections from 11 necropsies from individuals with pulmonary TB were used. Only in two cases mycobacteria were isolated from sputum, both were drug sensitive and they were not genotyped.
